# A Novel Polyamine Oxidase from *Kluyveromyces marxianus* with Potential for Total Polyamine Determination

**DOI:** 10.3390/biotech15030068

**Published:** 2026-08-15

**Authors:** Egor P. Sergeev, Denis L. Atroshenko

**Affiliations:** 1 Department of Chemistry, Lomonosov Moscow State University, 119991 Moscow, Russia; egor.sergeev@chemistry.msu.ru; 2 Laboratory of Molecular Engineering, Federal Research Centre “Fundamentals of Biotechnology” RAS, 119071 Moscow, Russia; 3 Institute of Medicine, RUDN University, 117198 Moscow, Russia

**Keywords:** polyamine oxidase, *Kluyveromyces marxianus*, polyamines, acetylated polyamines, enzymatic assay, recombinant expression

## Abstract

Polyamines and their acetylated derivatives are promising biomarkers for noninvasive cancer diagnostics, creating demand for robust enzymatic tools for their detection. In this study, we screened several phylogenetically diverse yeast polyamine oxidases and identified a new enzyme from *Kluyveromyces marxianus* (KmaPAO) as a promising candidate for analytical development. Among the tested candidates, only enzymes from *K. marxianus* and *Lachancea thermotolerans* were obtained in soluble active form, while only KmaPAO could be purified and characterized in detail. KmaPAO was produced in soluble active form in *Escherichia coli* at approximately 250 ± 40 mg of active enzyme per liter of culture, purified to near homogeneity in a single IMAC step, and showed a melting temperature of 66.6 ± 0.5 °C. The enzyme preferred acetylated polyamines and spermine, while showing lower activity toward spermidine. Kinetic analysis revealed sub-micromolar or low-micromolar Michaelis constants for several substrates and the highest catalytic efficiency toward spermine, 2.2 × 10^7^ M^−1^ s^−1^. Due to its favorable expression level, stability, and substrate profile, KmaPAO represents a promising basis for the development of enzymatic assays for total polyamine determination.

## 1. Introduction

Polyamines (putrescine, cadaverine, spermidine and spermine) and their acetylated derivatives are involved in the regulation of the cell cycle and cell proliferation [1]. There are a number of reports indicating that concentrations of different polyamines, particularly N^1,12^-diacetylspermine, are changed in saliva and urine in patients with different types of cancer [2,3,4,5]. Current methods for polyamine determination mainly rely on chromatographic and mass spectrometric approaches [6,7,8]. Although these methods are highly informative and sensitive, they require expensive equipment, specialized sample preparation, and trained personnel. Therefore, simpler enzymatic assays with colorimetric, fluorometric, or electrochemical detection remain attractive for targeted and potentially more accessible polyamine determination. One promising approach is the use of polyamine oxidases as analytical enzymes.

Polyamine oxidase is one of key enzymes of polyamine catabolism. Today, many different polyamine oxidases from plants [9], mammals and fungi [10,11,12,13] have been characterized, and several polyamine oxidase- or amine oxidase-based assays have previously been developed for the enzymatic determination of total or differential levels of diamines, spermidine, and spermine in biological samples [14,15,16,17,18]. However, these earlier approaches were mainly focused on non-acetylated polyamines and did not specifically evaluate the contribution of acetylated polyamine derivatives, although the levels of these compounds may substantially change in biological fluids. In addition, many previously described enzymes or assay systems have limitations for further biotechnological and analytical development, including the use of natural enzyme preparations, combinations of different amine oxidases, limited substrate specificity, or insufficient information on scalable production and storage stability. More recently, a recombinant polyamine oxidase from *Debaryomyces hansenii* was proposed for the determination of urinary diacetylspermine [19]. In our previous work, we cloned and characterized two polyamine oxidases with promising properties for detection of total acetylated polyamine content [13].

This article describes another polyamine oxidase from thermotolerant yeast, *K. marxianus*. After rigorous bioinformatic analysis [13], we noticed that there are a number of phylogenetically diverse branches of yeast enzymes with low sequence identity. We have chosen different yeasts from *Saccharomycetales*, *Phaffomycetales* and *Sporopachydermiales* in order to obtain polyamine oxidases with a substrate scope different from those already studied. *Kluyveromyces marxianus*, *Lachancea thermotolerans*, *Kazachstania bovina*, *Cyberlindnera jadinii* and *Sporopachydermia lactativora* genes were chosen for this study. Polyamine oxidase from *K. marxianus* (KmaPAO) and from *L. thermotolerans* (LtePAO) were expressed in soluble and active forms. The yield of *L. thermotolerans* and purity after IMAC were insufficient for subsequent work. We purified KmaPAO to homogeneity and examined the influence of temperature and pH on enzyme activity and stability as well as kinetic parameters toward polyamines. KmaPAO shows preference for acetylated polyamines and spermine, but is less active toward spermidine. Based on the high protein yield from culture and wide substrate scope, this enzyme could be a basis for the measurement of total polyamine content.

## 2. Materials and Methods

### 2.1. Materials

*K. marxianus* CBS 6556 and *L. thermotolerans* CBS 6340 were received from the Russian National Collection of Industrial Microorganisms (VKPM, Moscow, Russia) whereas *K. bovina* Y-1021, *S. lactativora* CBS 5771 and *C. jadinii* CBS 621 were received from the All-Russian Collection of Microorganisms (VKM, Pushchino, Moscow Region, Russia). *E. coli* Top10 cells were used for cloning and *E. coli* ArcticExpress (DE3) for protein expression. N^1^-acetylspermidine (N^1^-AcSpd), N^8^-acetylspermidine (N^8^-AcSpd), N^1,8^-diacetylspermidine (N^1,8^-AcSpd), N^1^-acetylspermine (N^1^-AcSpm) and N^1,12^-diacetylspermine (N^1,12^-AcSpm) were synthesized in a previous study [13]. Spermine (Spm), spermidine (Spd), putrescine (Put) and 1,3-diaminopropane (DAP) were purchased from Shanghai Macklin Biochemical Technology Co., Ltd. (Shanghai, China). Horseradish peroxidase was obtained from Dia-M (Moscow, Russia), and 10-acetyl-3,7-dihydroxyphenoxazine (Amplex Red) was obtained from Angene International Limited (Nanjing, China). Restriction and DNA modifying enzymes were purchased from SibEnzyme Ltd. (Novosibirsk, Russia). Oligonucleotides were synthesized by Lumiprobe RUS Ltd. (Moscow, Russia). Other chemicals were purchased from Sigma-Aldrich (St. Louis, MO, USA), Panreac Química SLU (Castellar del Vallès, Barcelona, Spain), and Merck KGaA (Darmstadt, Germany).

### 2.2. Construction of the Bacterial Expression Vector

Enzyme-coding DNA (accession codes in Table S1) were produced by PCR using the purified genomic DNA [20] as a template and the respective primer pairs (Table S1). PCR amplification was performed using a QuantStudio 5 Real-Time PCR System (Applied Biosystems, Thermo Fisher Scientific, Waltham, MA, USA). The resultant PCR product was cloned into the CloneJet PCR cloning kit and subcloned into the pET-24a (Novagen, Madison, WI, USA) via the NdeI and XhoI restriction sites. For quality control of DNA samples during cloning procedures NanoDrop One^C^ spectrophotometer (Thermo Scientific, Wilmington, DE, USA) was used. Sequences were verified by the ABI PRISM^®^ BigDye™ Terminator v. 3.1 Cycle Sequencing Kit (Applied Biosystems, Foster City, CA, USA), followed by analysis of the reaction products on a 3730 DNA Analyzer (Applied Biosystems, Foster City, CA, USA).

### 2.3. Expression and Purification of Polyamine Oxidases

The expression vectors were transformed into *E. coli* ArcticExpress (DE3) competent cells. The overnight culture was inoculated in a ratio of 1:100 into a 1 L baffled Erlenmeyer flask with 200 mL of 2YT medium with phosphates, 10 mL/L glycerol, and 30 mg/L kanamycin and induced with 0.1 mM isopropyl-*β*-D-thiogalactopyranoside at OD_600_ = 1.0–1.2. Cells were grown for 60 h at 18 °C.

After incubation, the cells were harvested by centrifugation at 4500× *g* for 5 min at 4 °C and resuspended in 50 mM sodium phosphate, 150 mM NaCl, and pH 7.5. After one freeze–thaw cycle, cells obtained from 200 mL of culture were disrupted by sonication using a Sonifier 250 ultrasonic homogenizer (Branson Ultrasonics Corporation, Danbury, CT, USA) for five 30 s cycles at power setting 5 in 50% pulse mode, with 30 s cooling intervals between cycles. The crude extract was clarified by centrifugation at 12,000× *g*, and the supernatant was loaded onto a 2 mL column packed with AbiFlow 100 Ni-AZUR Agarose (Abisense LLC, Sirius, Russia) equilibrated with the same buffer. The column was washed with the same buffer containing 25 mM imidazole until baseline absorbance and the protein was eluted with a 250 mM imidazole in 5 column volumes of the same buffer. Fractions containing KmaPAO were pooled and desalted with the help of Sephadex G-25 resin (Pharmacia AB, Uppsala, Sweden).

### 2.4. SDS-PAGE and Analytical Gel Filtration

This assay was performed similarly to the method described previously [13,21], using Precision Plus Protein Unstained Standards (Bio-Rad Laboratories, Inc., Hercules, CA, USA).

### 2.5. Determination of PAO Enzyme Activity

PAO enzyme activity was determined by a continuous measuring hydrogen peroxide generation using the peroxidase-coupled method with Amplex Red. All experiments were carried out in 50 mM Tris-HCl buffer, pH 9.0, at 25 °C with 0.1 mM spermine unless otherwise specified. The accumulation of the oxidized AmplexRed product was detected spectrophotometrically at 570 nm with an extinction coefficient of 64,350 M^−1^ cm^−1^ (Shimadzu Corporation, Kyoto, Japan). The extinction coefficient in this buffer was measured using a peroxidase reaction with a certain concentration of H_2_O_2_, which was determined spectrophotometrically (ε (240 nm) = 43.6 M^−1^ cm^−1^). For units (U) of enzyme activity, we used the amount of enzyme that is required for conversion of 1 micromole of substrate per 1 min (standard international units).

### 2.6. Determination of Products of Enzymatic Reaction

The amine products formed during KmaPAO-catalyzed oxidation of polyamines were identified by HPLC with fluorescence detection after dansyl chloride derivatization and comparison with authentic amine standards, as described previously [13].

### 2.7. Thermal Activity and Stability

To determine the dependence of PAO enzyme activity on temperature, activities were measured at the corresponding temperatures.

For temperature stability experiments, 0.1 mL of each enzyme solution at a concentration of 100 μg/mL in 100 mM sodium phosphate buffer with pH 7.0 was placed in thin-walled 0.5 mL Eppendorf tubes. The tubes were incubated in a water thermostat; after 10 min they were removed and immediately cooled on ice and then the activity was measured. The temperature was increased in 5 °C steps for activity experiments and in 2–3 °C steps for the stability experiment. Melting temperature (T_m_) was determined with a ThermoFAD assay [22] using the QuantStudio 5 Real-Time PCR system.

### 2.8. pH Activity and Stability

For pH activity experiments, enzyme solutions were diluted with 50 mM phosphate-Tris-carbonate buffer with different pH values, and activity was measured in the same buffer. For pH stability experiments, residual activity was measured after incubation of the enzyme solutions (100 μg/mL) for 10 min at 58 °C in 50 mM phosphate-Tris-carbonate buffer.

### 2.9. Kinetic Parameter Determination

Initial rates were determined using a continuous fluorescence assay by measuring the increase in fluorescence intensity of the product of Amplex Red oxidation with a CLARIOstar^®^ Plus plate reader (BMG LABTECH GmbH, Ortenberg, Germany) (excitation at 570 nm; emission at 605 nm; fluorescence readings every 10 s). The H_2_O_2_ generation rate was calculated from the change in fluorescence intensity, using calibration curves obtained by serial dilutions of a stock solution of H_2_O_2_. Steady state kinetic parameters (V_max_ and K_m_) were calculated by nonlinear regression using OriginPro 9.1 (OriginLab Corporation, Northampton, MA, USA). Active-site concentration for *k_cat_* determination was estimated from the absorbance of FAD using the FAD extinction coefficient immediately after desalting. Enzyme activity remained unchanged within the experimental error throughout the period of experimental work.

## 3. Results and Discussion

We performed a rigorous bioinformatic analysis in our previous study [13]. It showed that yeast PAOs are divided into two major clades, one whose common ancestor most likely had peroxisomal targeting signal type 1 (PTS1) and one with a common ancestor without PTS1. In this study we choose sequences from the second clade, from species which are evolutionary distant from the only characterized enzyme of this branch—Fms1 from *Saccharomyces cerevisiae* [10]. Species belonging to *Serinales* were excluded because their alternative genetic code complicates cloning from genomic DNA and recombinant expression in *E. coli* [23]. Also, we selected only thermotolerant yeast for greater thermostability potential. Based on these criteria, we used genes from *K. marxianus* CBS 6556, *L. thermotolerans* CBS 6340, *K. bovina* Y-1021, *S. lactativora* CBS 5771 and *C. jadinii* CBS 621 in the study.

**Figure 1:**
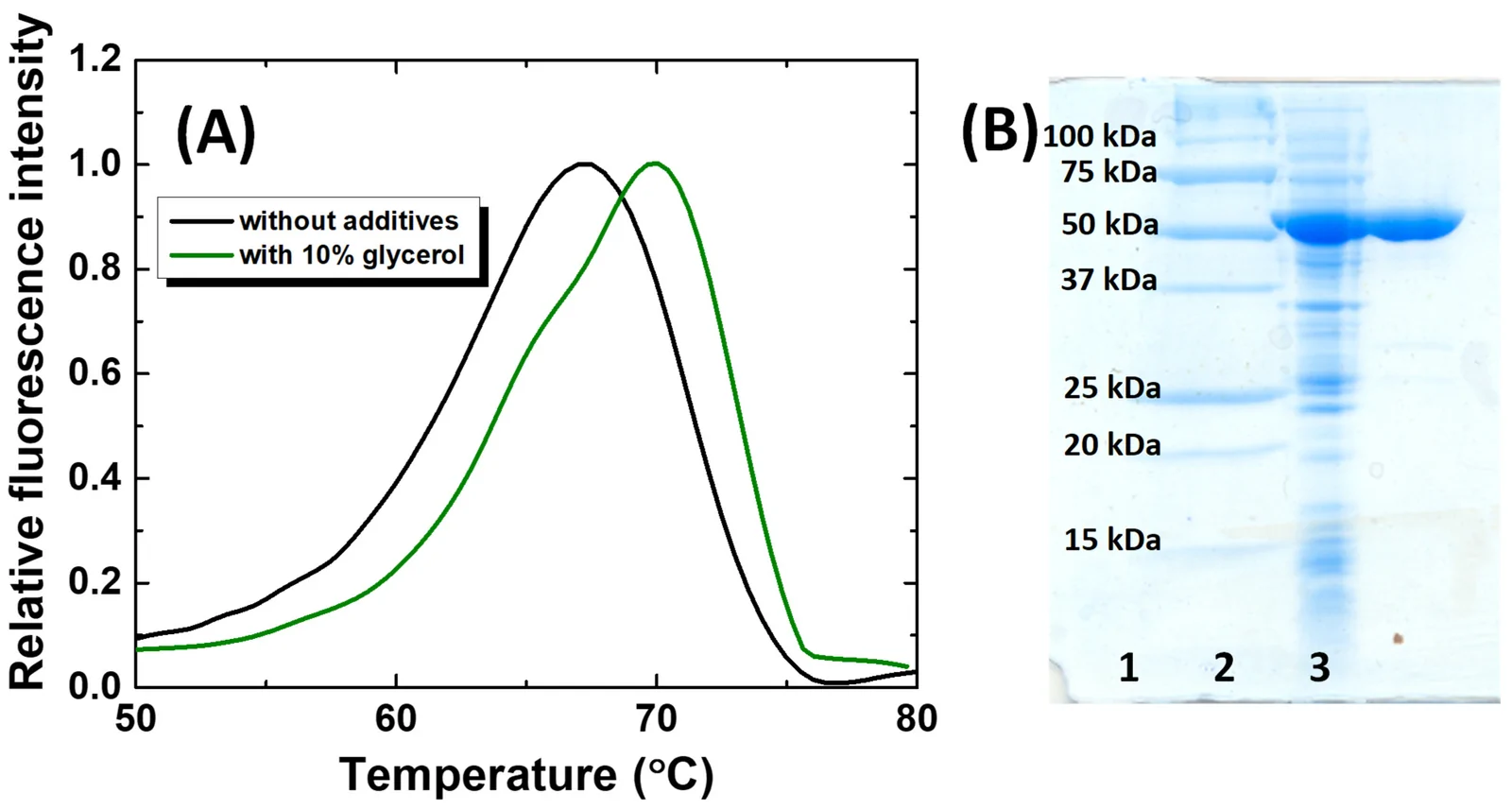
ThermoFAD analysis and purification of KmaPAO. (**A**) ThermoFAD profiles of KmaPAO recorded in the absence and presence of glycerol. (**B**) SDS-PAGE analysis of KmaPAO expression and purification: lane 1, Precision Plus Protein Unstained Standards (Bio-Rad); lane 2, crude *E. coli* lysate expressing KmaPAO; lane 3, purified KmaPAO.

All genes potentially encoding polyamine oxidase were cloned into the pET-24a plasmid with a vector-encoded C-terminal His-tag through NdeI and XhoI restriction sites. In case of KmaPAO and LtePAO, an XhoI-compatible sticky end was mimicked with the BsaI restriction enzyme. For soluble expression of FAD-dependent oxidases we used the ArcticExpress (DE3) strain of *E. coli*. Only KmaPAO and LtePAO showed activity in crude extract after culturing. One-step purification of KmaPAO was enough for more than 95% purity (Figure 1B) but LtePAO purification showed unfavorable results. For subsequent work only KmaPAO was used as it was more robust. The level of expression for KmaPAO was about 600 ± 100 U with spermine per liter of culture medium, which corresponds to 250 ± 40 mg of active enzyme per liter. Yield after IMAC purification was 30 ± 10%.

**Figure 2:**
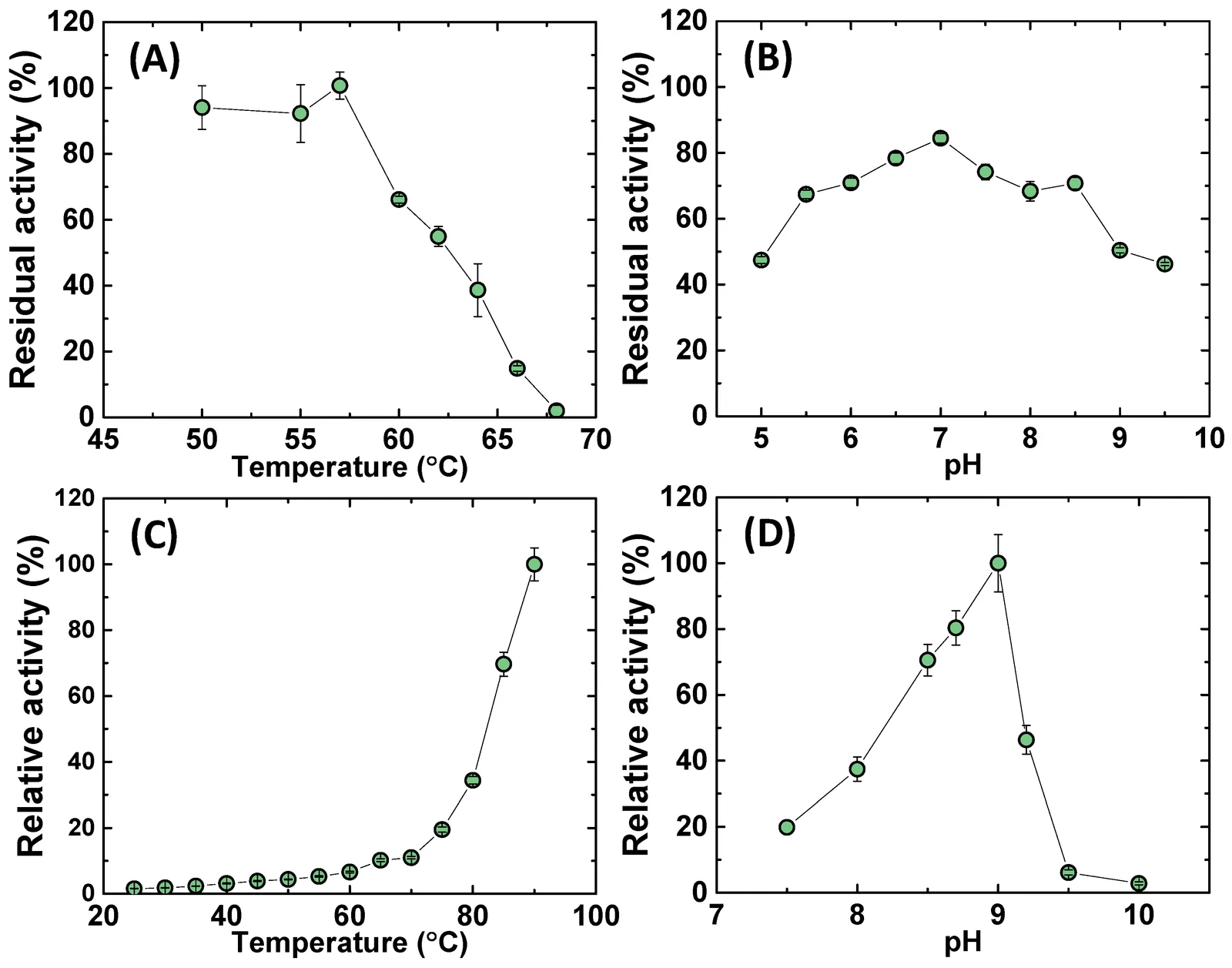
Activity and stability of KmaPAO at different pH and temperatures. Error bars represent standard deviation (SD), *n* = 3. For stability experiments (**A**,**B**), residual activity was measured under standard assay conditions: 50 mM Tris-HCl buffer, pH 9.0, 25 °C, with 0.1 mM Spm. (**A**) Temperature stability profile, residual activity after incubation of 0.1 mg/mL of enzyme for 10 min in 0.1 M sodium phosphate buffer, pH 7.0 at the indicated temperatures. Here, 100% activity corresponds to 2.4 ± 0.2 U/mg. (**B**) pH stability profile; residual activity after incubation of 0.1 mg/mL of enzyme in phosphate-Tris-carbonate buffer (50 mM each) at the indicated pH values for 10 min at 58 °C. Residual activity is expressed relative to the initial activity before incubation; 100% corresponds to 2.4 ± 0.2 U/mg. (**C**) Dependence of activity on temperature, measured at the indicated temperatures in 50 mM Tris-HCl buffer, pH 9.0, with 0.1 mM Spm. Activity is expressed relative to the maximum value measured in this experiment. (**D**) pH activity profile; activity measured in phosphate-Tris-carbonate buffer (50 mM each) at the indicated pH values at 25 °C with 0.1 mM Spm. Activity is expressed relative to the maximum value measured in this experiment.

The enzyme showed the best stability at pH 7.0 (Figure 2). The highest activity toward spermine was observed at pH 9.0, which is the same as for other enzymes from yeasts and fungi. However, our enzyme shows a sharp decline in activity at a pH greater than 9, which is not typical for other representatives of this class. In the temperature-dependence assay performed with spermine as the substrate, KmaPAO showed a strong increase in apparent activity at elevated temperatures and retained measurable activity even at 90 °C during the short assay time. The activity at 90 °C was approximately 6500 ± 300% of that observed at 25 °C. This observation may reflect a spermine-associated protective effect under the assay conditions. The enzyme oxidizes the C-N bond closest to acetylated amine for acetylated derivatives and the C–N bond toward the C3 fragment in non-acetylated polyamines (Table 1). The apparent molecular weight of KmaPAO estimated by gel filtration chromatography was 130 ± 20 kDa, which roughly corresponds to a dimeric form.

For optimization of storage conditions, we tested glycerol (10%, 20%), polyethylene glycol 6000 (5%), Triton X-100 (0.01%, 0.1%), Tween 20 (0.1%, 0.2%) and EDTA (0.2 mM) using the ThermoFAD assay. The greatest improvement was achieved with 10% glycerol, which shifted the melting temperature of KmaPAO from 66.6 ± 0.5 °C to 69.9 ± 0.2 °C (Figure 1A). EDTA did not change the melting temperature, and was added to the storage buffer for bacterial growth inhibition. The enzyme could be stored at −20 °C in frozen form for 6 months and lost about 10% of its activity after a freeze–thaw cycle, while at +4 °C the enzyme lost 20% of activity within a week.

The maximum reaction rate (V_max_) for Spm is 2.52 ± 0.07 U/mg, for Spd 0.60 ± 0.02 U/mg, for N^1^-AcSpd 2 ± 0.1 U/mg, for N^8^-AcSpd 2.0 ± 0.2 U/mg, for N^1^-AcSpm 1.31 ± 0.03 U/mg, and for N^1,12^-AcSpm 4.8 ± 0.2 U/mg. The obtained catalytic parameters show extremely low Michaelis constants (Table 1, Figures S2–S7). The catalytic efficiencies (*k_cat_*/K_M_) for Spm, N_1_-AcSpm, and N^1,12^-AcSpm were markedly higher than those for Spd and its acetylated derivatives and were comparable to one another. Overall, these values were within the same range as those reported for the enzyme from *Saccharomyces cerevisiae* [10]. Catalytic parameters reported for other PAOs toward their preferred substrates are summarized in Table S2. Compared with other yeast PAOs, KmaPAO generally exhibited lower *k_cat_* and K_M_ in comparison with other yeast enzymes. The PAO from *D. hansenii* is highly specific for N^1,12^-AcSpm [19]. Enzymes from *O. parapolymorpha* are unable to oxidize Spm or Spd [13]. Human and mouse spermine oxidases show strong preference for Spm: the mouse enzyme is approximately 1000-fold more active toward spermine than toward the closely related substrate N^1^-AcSpm [24], and the V_max_/K_M_ value for Spm is also approximately 1000-fold higher than that for N^1^-AcSpm in the human enzyme [25]. Human PAO is inactive toward Spm, Spd, and N^8^-AcSpd [26], while mouse PAO is inactive toward Spd and N^8^-AcSpd [27]. Plant enzymes are generally specific toward non-acetylated polyamines [28]. Earlier PAO-based assays using plant or fungal enzymes demonstrated the feasibility of enzymatic polyamine determination, but they were mainly focused on non-acetylated polyamines and often relied on natural enzyme preparations or combinations of different amine oxidases [14,15,16,17,18].

In this context, KmaPAO appears promising for future assays aimed at total polyamine determination because it combines activity toward Spm, Spd, and the major acetylated polyamines with soluble recombinant production in *E. coli*, straightforward purification, good stability, and low Michaelis constants for several substrates. The present biochemical characterization provides a basis for further analytical development of KmaPAO-based assays, which will require separate validation in real biological matrices, including saliva and urine, with assessment of matrix effects, recovery, linearity, detection limits, and reproducibility.

## 4. Conclusions

In this work, we identified and characterized a new yeast polyamine oxidase from *Kluyveromyces marxianus*. Among the tested candidates, KmaPAO was the most promising enzyme because it could be produced in soluble active form in *Escherichia coli*, purified with good yield, and characterized in detail. KmaPAO showed broad substrate scope, preference for acetylated polyamines and spermine, very low Michaelis constants for several substrates, and good stability. These properties make KmaPAO a promising candidate for the development of enzymatic assays for total polyamine determination.

## Figures and Tables

**Table 1:** Steady state kinetic parameters of KmaPAO. All measurements were performed in the range from 0.5 to 10 K_M_ with the corresponding substrate in 50 mM TrisHCl at pH 9.0 at 25 °C. Values are presented as fitted parameter ± standard error (SE) obtained from nonlinear regression.

Substrate	*k_cat_*, s^−1^	K_M_, μM	*k_cat_*/K_M_, s^−1^ M^−1^	Amine Product
Spm	2.40 ± 0.06	0.11 ± 0.02	2.2 × 10^7^	Spd
N^1^-AcSpm	1.25 ± 0.03	0.070 ± 0.005	1.8 × 10^7^	Spd
N^1,12^-AcSpm	4.6 ± 0.2	0.24 ± 0.04	1.9 × 10^7^	Put
Spd	0.6 ± 0.2	5.1 ± 0.4	1.1 × 10^5^	Put with minor amounts of DAP
N^1^-AcSpd	1.9 ± 0.1	0.22 ± 0.04	8.7 × 10^6^	Put
N^8^-AcSpd	1.9 ± 0.2	2.3 ± 0.6	8.3 × 10^5^	DAP

## Data Availability

The original contributions presented in this study are included in the article/Supplementary Materials. Further inquiries can be directed to the corresponding authors.

## Supplementary Materials

The following supporting information can be downloaded at: https://www.mdpi.com/article/10.3390/biotech15030068/s1, Table S1: List of oligonucleotides and gene identifiers used in the study. UniProt identifiers were used for KmaPAO, LtePAO, and CjaPAO; the NCBI Protein identifier is indicated for KboPAO. The SlaPAO coding sequence was identified in an unannotated region of *Sporopachydermia lactativora* NRRL Y-11591 WGS contig JAKTRH010000040.1, coordinates 12,576–13,994, on the plus strand. Table S2: Catalytic parameters of polyamine oxidases from various organisms toward their preferred substrates. Figure S1: Multiple sequence alignment of previously characterized yeast polyamine oxidases and the proteins analyzed in this study. Figure S2: Michaelis–Menten kinetics of KmaPAO with spermine as the substrate. Initial reaction rates were measured at different spermine concentrations. The final KmaPAO concentration in the reaction mixture was 4.2 ng/mL. The experimental data were fitted to the Michaelis–Menten equation. Open circles represent the experimental values, and the solid line represents the nonlinear regression fit. Figure S3: Michaelis–Menten kinetics of KmaPAO with N^1^-acetylspermine as the substrate. Initial reaction rates were measured at different N^1^-acetylspermine concentrations. The final KmaPAO concentration in the reaction mixture was 4.2 ng/mL. The experimental data were fitted to the Michaelis–Menten equation. Open circles represent the experimental values, and the solid line represents the nonlinear regression fit. Figure S4: Michaelis–Menten kinetics of KmaPAO with N^1,12^-diacetylspermine as the substrate. Initial reaction rates were measured at different N^1,12^-diacetylspermine concentrations. The final KmaPAO concentration in the reaction mixture was 4.2 ng/mL. The experimental data were fitted to the Michaelis–Menten equation. Open circles represent the experimental values, and the solid line represents the nonlinear regression fit. Figure S5: Michaelis–Menten kinetics of KmaPAO with spermidine as the substrate. Initial reaction rates were measured at different spermidine concentrations. The final KmaPAO concentration in the reaction mixture was 4.2 ng/mL. The experimental data were fitted to the Michaelis–Menten equation. Open circles represent the experimental values, and the solid line represents the nonlinear regression fit. Figure S6: Michaelis–Menten kinetics of KmaPAO with N^1^-acetylspermidine as the substrate. Initial reaction rates were measured at different N^1^-acetylspermidine concentrations. The final KmaPAO concentration in the reaction mixture was 375 ng/mL. The experimental data were fitted to the Michaelis–Menten equation. Open circles represent the experimental values, and the solid line represents the nonlinear regression fit. Figure S7: Michaelis–Menten kinetics of KmaPAO with N^8^-acetylspermidine as the substrate. Initial reaction rates were measured at different N^8^-acetylspermidine concentrations. The final KmaPAO concentration in the reaction mixture was 4.2 ng/mL. The experimental data were fitted to the Michaelis–Menten equation. Open circles represent the experimental values, and the solid line represents the nonlinear regression fit.

## Abbreviations

The following abbreviations are used in this manuscript:

PAO	polyamine oxidase
KmaPAO	polyamine oxidase from *Kluyveromyces marxianus*
LtePAO	polyamine oxidase from *Lachancea thermotolerans*
IMAC	immobilized metal affinity chromatography
SDS-PAGE	sodium dodecyl sulfate–polyacrylamide gel electrophoresis
PCR	polymerase chain reaction
EDTA	ethylenediaminetetraacetic acid
PEG	polyethylene glycol
Spm	spermine
Spd	spermidine
Put	putrescine
DAP	1,3-diaminopropane
N^1^-AcSpd	N^1^-acetylspermidine
N^8^-AcSpd	N^8^-acetylspermidine
N^1,8^-AcSpd	N^1,8^-diacetylspermidine
N^1^-AcSpm	N^1^-acetylspermine
N^1,12^-AcSpm	N^1,12^-diacetylspermine
Tm	melting temperature
PTS1	peroxisomal targeting signal type 1
