# Supplementary material for: A Novel Polyamine Oxidase from Kluyveromyces marxianus with Potential for Total Polyamine Determination
Source: BioTech (Basel). 2026 Aug 15;15(3):68. doi: 10.3390/biotech15030068 (PMC13510455; doi:10.3390/biotech15030068)
Supplement: Supplementary file 1 [file biotech-15-00068-s001.zip › biotech-4291825-supplementary.pdf]

BioTech MDPI

Supplementary Materials: A novel polyamine oxidase from *Kluyveromyces marxianus* with potential for total polyamine determination

Egor P. Sergeev and Denis L. Atroshenko

Table S1. List of oligonucleotides and gene identifiers used in the study. UniProt identifiers were used for KmaPAO, LtePAO, and CjaPAO; the NCBI Protein identifier is indicated for KboPAO. The SlaPAO coding sequence was identified in an unannotated region of the *Sporopachydermia lactativora* NRRL Y-11591 WGS contig JAKTRH010000040.1, coordinates 12576–13994, on the plus strand.

Oligonucleotide name	Oligonucleotide sequence	Corresponding gene
KmaPAO, For	AGCTTCA**CAT*ATG****TCCACCCCCACGCAC*	A0ABX6EWL5
KmaPAO, Rev	TGACT**GGTCTC**CTCGAG*TCTTGTAAATACTTT-GTCAAGGATATACTTAGC*	A0ABX6EWL5
LtePAO, For	AGCTTCA**CAT*ATG****ATTGAAAACCAGGTGG-TAATTATTGG*	C5DGS8
LtePAO, Rev	TGACT**GGTCTC**CTCGAGAG*CGAGTTTATCAGCGA-TAAAATCTGC*	C5DGS8
SlaPAO, For	CGACCT**CAT*ATG****GCTGCTAGTATTTTCAAACACCG*	JAKTRH010000040.1:12576–13994 (+)
SlaPAO, Rev	AATAAT**CTCGAG***TCCCCTCCTTACAGACCTCC*	JAKTRH010000040.1:12576–13994 (+)
CjaPAO, For	CGACCT**CAT*ATG****CCTGTGGATACAAAAGTTGTG*	A0A1E4SY1
CjaPAO, Rev	CGACGT**CTCGAG***ACCAGCAACGTGGTTGATGATA-TAAG*	A0A1E4SY1
KboPAO, For	CGACCT**CAT*atg****atgtccagagtagtgattatcgg*	XP_064716516
KboPAO, Rev	AGGTCG**CTCGAG**ATTTTCGGCGATA-TAAATGGCTTCACG	XP_064716516

Table S2. Catalytic parameters of polyamine oxidases from various organisms toward their preferred substrates.

Organism	Enzyme	Substrate with highest k_{cat}/K_M	Catalytic rate*	K_M, mkM	k_{cat} / K_M, $M^{-1}s^{-1}$	DOI
Sacharamyces cerevisiae	FMS1	Spm	165 s^{-1}	10	$1.65*10^7$	10.1016/s0006-291x(03)00416-9
Debaryomyces hansenii T-42	DASpmOx	$N^{1,12}$-AcSpm	147 s^{-1}	83	$1.77*10^6$	10.1016/j.bbapap.2007.08.010
Ogataeaa parapolymorpha DL-1	OpaPAO1	N^1-AcSpm	200±30 s^{-1}	1.0±0.3	$2*10^8$	10.1515/hsz-2025-0158
	OpaPAO3	N^1-AcSpm	6.2±0.2 s^{-1}	2.6±0.4	$2.4*10^6$	
Human	hSMO	Spm	6.6±0.2 s^{-1}	190±32	$37±7*10^3$	10.1021/bi9017945
	hPAOx	N^1-AcSpm	31.7 s^{-1}	0.85	$3.72*10^7$	10.1007/s00280-004-0936-5
Mouse	mSMO	Spm	4.5 s^{-1}	90	$5*10^4$	10.1074/jbc.m207888200
	mPAOX	N^1-AcSpm	4.5 s^{-1}	1.8	$2.5*10^6$	10.1074/jbc.M302149200
Arabidopsis thaliana	AtPAO1	NorSpm	6.9±1.3 s^{-1}	90±10	$81.4*10^3$	10.1104/pp.106.080911
	AtPAO2	Spm	4.2±1.2 s^{-1}	270±30	$1.55*10^4$	10.1093/jxb/erq341
	AtPAO3	Spd	1.25 s^{-1}	204	$6.12*10^3$	10.1104/pp.108.123802
	AtPAO4	Spm	1.4 s^{-1}	230	$6.1*10^3$	10.1093/pcp/pcn114
	AtPAO5	Spm (pH 7.5)	0.069 s^{-1}	25.56	$2.7*10^3$	10.1104/pp.114.242610
Maize	maPAO	Spd	50.2±6.3 s^{-1}	2.1±0.3	$23.9*10^6$	10.1021/bi050983i
Branchiostoma japonicum	BjPAO1	Spd	7.84 U/mg	7		10.1016/j.gene.2015.09.017
	BjPAO2	Spd	10 U/mg	12.36		10.1016/j.gene.2015.09.017
Acanthamoeba culbertsoni	AcuPAO	N^8-AcSpd	5.6 U/mg	105±13		10.1016/0166-6851(92)90204-W

Synechocystis sp. PCC 6803	SyPAO	Spm	0.31±0.01 s^{-1}	98±10	(3.16±0.2)*10^3	10.1016/j.plaphy.2017.08.018
Ascaris suum	AsuPAO	Spm	4.2 U/mg	350		10.1042/bj2830075
Oryza sativa	OsPAO1	ThSpm (pH 6.0)	0.051±0.002 s^{-1}	3.7±0.23	13.688*10^3	10.1007/s00299-013-1518-y
	OsPAO7	Spm	30.6±0.5 s^{-1}	40.7±1.2	(0.76±0.014)*10^6	10.1093/pcp/pcu047

*Catalytic rate is presented as k_{cat} (s^{-1}) when available or as maximum specific activity (U/mg) when k_{cat} was not reported.

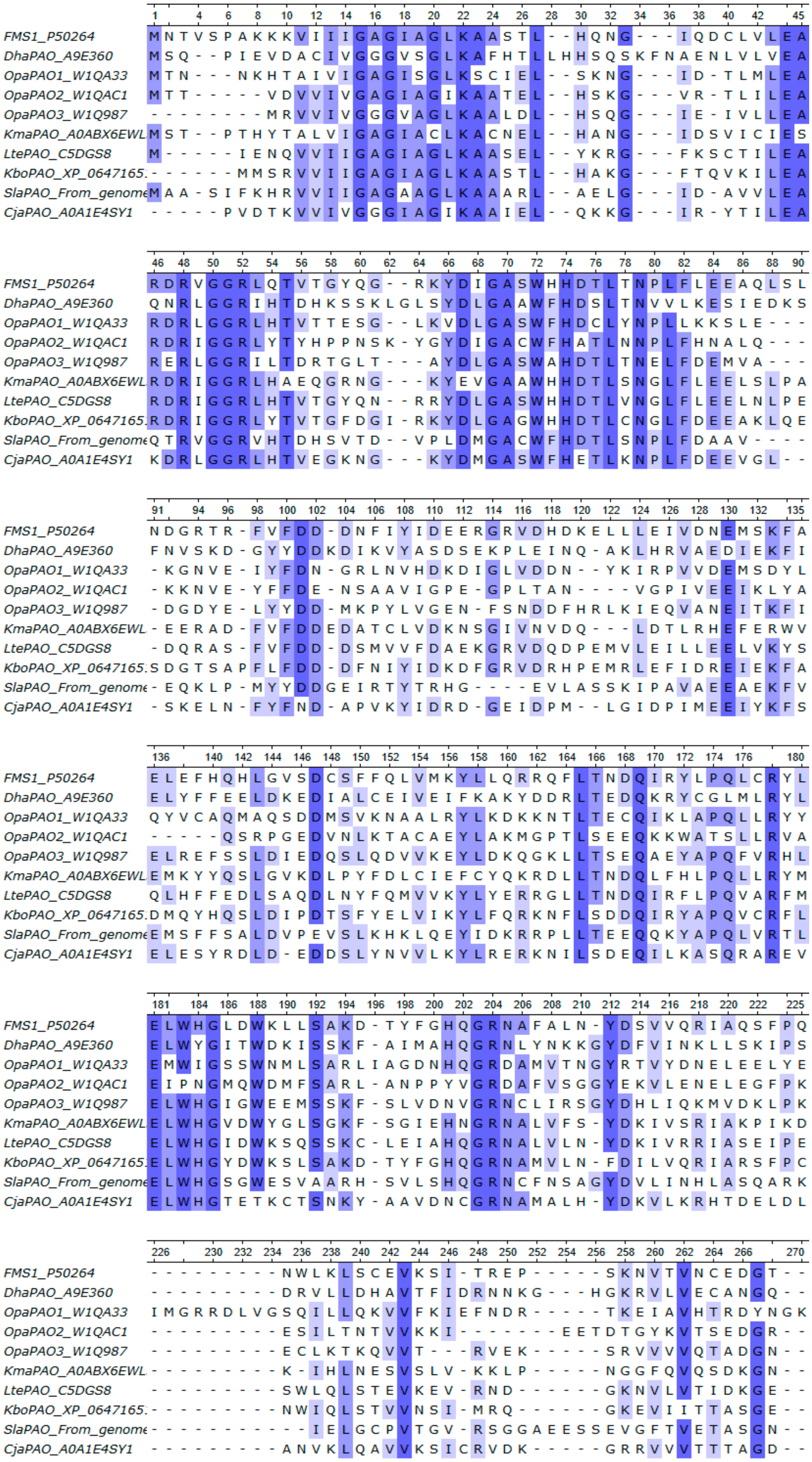
1 4 6 8 10 12 14 16 18 20 22 24 26 28 30 32 34 36 38 40 42 45
FMS1_P50264 MNTVSPAKKKVIIIGAGIAGLKAASTL--HQNG---IQDCLVLEA
DhaPAO_A9E360 MSQ--PIEVDACIVGGGVSGLKAFHTLLHHSQSKFNAENLVLVEA
OpaPAO1_W1QA33 MTN---NKHTAIVIGAGISGLKSCIEL--SKNG---ID-TLMLEA
OpaPAO2_W1QAC1 MTT-----VDVVIVGAGIAGIKAATEL--HSKG---VR-TLILEA
OpaPAO3_W1Q987 -------MRVVIVGGGVAGLKAALDL--HSQG---IE-IVLLEA
KmaPAO_A0ABX6EWL MST--PTHYTALVIGAGIACLKACNEL--HANG---IDSVICIES
LtePAO_C5DGS8 M----IENQVVIIGAGIAGLKAASEL--YKRG---FKSCTILEA
KboPAO_XP_0647165. ------MMSRVVIIGAGIAGLKAASTL--HAKG---FTQVKILEA
SlaPAO_From_genome MAA-SIFKHRVVIIGAGAAGLKAAARL--AELG---ID-AVVLEA
CjaPAO_A0A1E4SY1 -----PVDTKVVIVGGGIAGIKAAIEL--QKKG---IR-YTILEA
46 48 50 52 54 56 58 60 62 64 66 68 70 72 74 76 78 80 82 84 86 88 90
FMS1_P50264 RDRVGGRLQTVTGYQG--RKYDIGASWHHDTLTNPLFLEEAQLSL
DhaPAO_A9E360 QNRLGGRIHTDHKSSKLGLSYDLGAAWFHDSLTNVVLKESIEDKS
OpaPAO1_W1QA33 RDRLGGRLHTVTTESG--LKVDLGASWFHDCLYNPLLKKSLE---
OpaPAO2_W1QAC1 RDRIGGRLYTYHPPNSK-YGYDIGACWFHATLNNPLFHNALQ---
OpaPAO3_W1Q987 RERLGGRILTDRTGLT---AYDLGASWAHDTLTNELFDEMVA---
KmaPAO_A0ABX6EWL RDRIGGRLHAEQGRNG---KYEVGAAWHHDTLSNGLFLEELSLPA
LtePAO_C5DGS8 RDRIGGRLHTVTGYQN--RRYDLGASWHHDTLVNGLFLEELNLPE
KboPAO_XP_0647165. RDRIGGRLYTVTGFDGI-RKYDLGAGWHHDTLCNGLFDEEAKLQE
SlaPAO_From_genome QTRVGGRVHTDHSVTD--VPLDMGACWFHDTLSNPLFDAAV----
CjaPAO_A0A1E4SY1 KDRLGGRLHTVEGKNG---KYDMGASWFHETLKNPLFDEEVGL--
91 94 96 98 100 102 104 106 108 110 112 114 116 118 120 122 124 126 128 130 132 135
FMS1_P50264 NDGRTR-FVFDD-DNFIYIDEERGRVDHDKELLLEIVDNEMSKFA
DhaPAO_A9E360 FNVSKD-GYYDDKDIKVYASDSEKPLEINQ-AKLHRVAEDIEKFI
OpaPAO1_W1QA33 -KGNVE-IYFDN-GRLNVHDKDIGLVDDN--YKIRPVVDEMSDYL
OpaPAO2_W1QAC1 -KKNVE-YFFDE-NSAAVIGPE-GPLTAN----VGPIVEEIKLYA
OpaPAO3_W1Q987 -DGDYE-LYYDD-MKPYLVGEN-FSNDDFHRLKIEQVANEITKFI
KmaPAO_A0ABX6EWL -EERAD-FVFDDEDATCLVDKNSGIVNVDQ---LDTLRHEFERWV
LtePAO_C5DGS8 -DQRAS-FVFDD-DSMVVFDAEKGRVDQDPEMVLEILLEELVKYS
KboPAO_XP_0647165. SDGTSAPFLFDD-DFNIYIDKDFGRVDRHPEMRLEFIDREIEKFA
SlaPAO_From_genome -EQKLP-MYYDDGEIRTYTRHG----EVLASSKIPAVAEEAEKFV
CjaPAO_A0A1E4SY1 -SKELN-FYFND-APVKYIDRD-GEIDPM--LGIDPIMEEIYKFS
136 140 142 144 146 148 150 152 154 156 158 160 162 164 166 168 170 172 174 176 180
FMS1_P50264 ELEFHQHLGVSDCSFFQLVMKYLLQRRQFLTNDQIRYLPQLCRYL
DhaPAO_A9E360 ELYFFEELDKEDIALCEIVEIFKAKYDDRLTEDQKRYCGLMLRYL
OpaPAO1_W1QA33 QYVCAQMAQSDDMSVKNAALRYLKDKKNTLTECQIKLAPQLLRYY
OpaPAO2_W1QAC1 -----QSRPGEDVNLKTACAEYLAKMGPTLSEEQKKWATSLLRVA
OpaPAO3_W1Q987 ELREFSSLDIEDQSLQDVVKEYLDKQGKLLTSEQAEYAPQFVRHL
KmaPAO_A0ABX6EWL EMKYYQSLGVKDLPYFDLCIEFCYQKRDLLTNDQLFHLPQLLRYM
LtePAO_C5DGS8 QLHFFEDLSAQDLNYFQMVVKYLYERRGLLTNDQIRFLPQVARFM
KboPAO_XP_0647165. DMQYHQSLDIPDTSFYELVIKYLFQRKNFLSDDQIRYAPQVCRFL
SlaPAO_From_genome EMSFFSALDVPEVSLKHKLQEYIDKRRPLLTEEQQKYAPQLVRTL
CjaPAO_A0A1E4SY1 ELESYRDLD-EDDSLYNVVLKYLRERKNILSDEQILKASQRAREV
181 184 186 188 190 192 194 196 198 200 202 204 206 208 210 212 214 216 218 220 222 225
FMS1_P50264 ELWHGLDWKLLSAKD-TYFGHQGRNAFALN-YDSVVQRIAQSFPQ
DhaPAO_A9E360 ELWYGITWDKISSKF-AIMAHQGRNLYNKKGYDFVINKLLSKIPS
OpaPAO1_W1QA33 EMWIGSSWNMLSARLIAGDNHQGRDAMVTNGYRTVYDNELEELYE
OpaPAO2_W1QAC1 EIPNGMQWDMFSARL-ANPPYVGRDAFVSGGYEKVLENELEGFPK
OpaPAO3_W1Q987 ELWHGIGWEEMSSKF-SLVDNVGRNCLIRSGYDHLIQKMVDKLPK
KmaPAO_A0ABX6EWL ELWHGVDWYGLSGKF-SGIEHNGRNALVFS-YDKIVSRIAKPIKD
LtePAO_C5DGS8 ELWHGIDWKSQSSKC-LEIAHQGRNALVLN-YDKIVRRIASEIPE
KboPAO_XP_0647165. ELWHGYDWKSLSAKD-TYFGHQGRNAMVLN-FDILVQRIARSFPC
SlaPAO_From_genome ELWHGSGWESVAARH-SVLSHQGRNCFNSAGYDVLINHLASQARK
CjaPAO_A0A1E4SY1 ELWHGTETKCTSNKY-AAVDNCGRNAMALH-YDKVLKRHTDELDL
226 230 232 234 236 238 240 242 244 246 248 250 252 254 256 258 260 262 264 266 270
FMS1_P50264 ---------NWLKLSCEVKSI-TREP-----SKNVTVNCEDGT--
DhaPAO_A9E360 ---------DRVLLDHAVTFIDRNNKG----HGKRVLVECANGQ--
OpaPAO1_W1QA33 IMGRRDLVGSQILLQKVVFKIEFNDR-----TKEIAVHTRDYNGK
OpaPAO2_W1QAC1 ---------ESILTNTVVKKI-------EETDTGYKVTSEDGR--
OpaPAO3_W1Q987 ---------ECLKTKQVVT---RVEK-----SRVVVVQTADGN--
KmaPAO_A0ABX6EWL ---------K-IHLNESVSLV-KKLP-----NGGFQVQSDKGN--
LtePAO_C5DGS8 ---------SWLQLSTEVKEV-RND------GKNVLVTIDKGE--
KboPAO_XP_0647165. ---------NWIQLSTVVNSI-MRQ------GKEVIITTASGE--
SlaPAO_From_genome -----------IELGCPVTGV-RSGGAEESSEVGFTVETASGN--
CjaPAO_A0A1E4SY1 ---------ANVKLQAVVKSICRVDK-----GRRVVVTTTAGD--

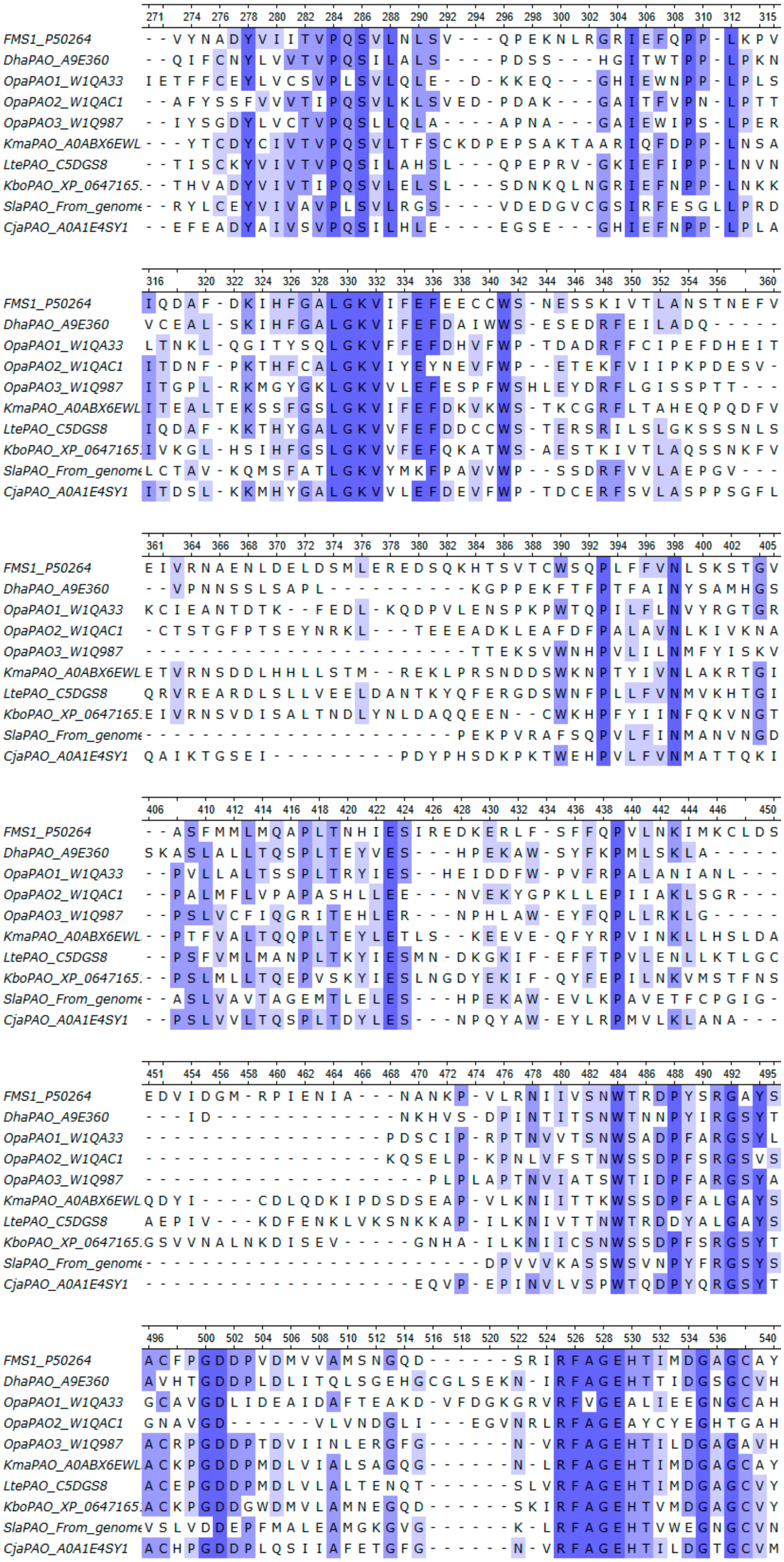
271 274 276 278 280 282 284 286 288 290 292 294 296 298 300 302 304 306 308 310 312 315
FMS1_P50264 --VYNADYVIITVPQSVLNLSV---QPEKNLRGRIEFQPP-LKPV
DhaPAO_A9E360 --QIFCNYLVVTVPQSILALS----PDSS---HGITWTPP-LPKN
OpaPAO1_W1QA33 IETFFCEYLVCSVPLSVLQLE--D-KKEQ---GHIEWNPP-LPLS
OpaPAO2_W1QAC1 --AFYSSFVVVTIPQSVLKLSVED-PDAK---GAITFVPN-LPTT
OpaPAO3_W1Q987 --IYSGDYLVCTVPQSLLQLA----APNA---GAIEWIPS-LPER
KmaPAO_A0ABX6EWL ---YTCDYCIVTVPQSVLTFSCKDPEPSAKTAARIQFDPP-LNSA
LtePAO_C5DGS8 --TISCKYVIVTVPQSILAHSL---QPEPRV-GKIEFIPP-LNVN
KboPAO_XP_0647165. --THVADYVIVTIPQSVLELSL---SDNKQLNGRIEFNPP-LNKK
SlaPAO_From_genome --RYLCEYVIVAVPLSVLRGS----VDEDGVCGSIRFESGLLPRD
CjaPAO_A0A1E4SY1 --EFEADYAIVSVPQSILHLE----EGSE---GHIEFNPP-LPLA
316 320 322 324 326 328 330 332 334 336 338 340 342 344 346 348 350 352 354 356 360
FMS1_P50264 IQDAF-DKIHFGALGKVIFEFEECCWS-NESSKIVTLANSTNEFV
DhaPAO_A9E360 VCEAL-SKIHFGALGKVIFEFDAIWWS-ESEDRFEILADQ-----
OpaPAO1_W1QA33 LTNKL-QGITYSQLGKVFFEFDHVFWP-TDADRFFCIPEFDHEIT
OpaPAO2_W1QAC1 ITDNF-PKTHFCALGKVIYEYNEVFWP--ETEKFVIIPKPDESV-
OpaPAO3_W1Q987 ITGPL-RKMGYGKLGKVVLEFESPFWSHLEYDRFLGISSPTT---
KmaPAO_A0ABX6EWL ITEALTEKSSFGSLGKVIFEFDKVKWS-TKCGRFLTAHEQPQDFV
LtePAO_C5DGS8 IQDAF-KKTHYGALGKVVFEFDDCCWS-TERSRILSLGKSSSNLS
KboPAO_XP_0647165. IVKGL-HSIHFGSLGKVVFEFQKATWS-AESTKIVTLAQSSNKFV
SlaPAO_From_genome LCTAV-KQMSFATLGKVYMKFPAVVWP--SSDRFVVLAEPGV---
CjaPAO_A0A1E4SY1 ITDSL-KKMHYGALGKVVLEFDEVFWP-TDCERFSVLASPPSGFL
361 364 366 368 370 372 374 376 378 380 382 384 386 388 390 392 394 396 398 400 402 405
FMS1_P50264 EIVRNAENLDELDSMLEREDSQKHTSVTCWSQPLFFVNLSKSTGV
DhaPAO_A9E360 --VPNNSSLSAPL----------KGPPEKFTFPTFAINYSAMHGS
OpaPAO1_W1QA33 KCIEANTDTK--FEDL-KQDPVLENSPKPWTQPILFLNVYRGTGR
OpaPAO2_W1QAC1 -CTSTGFPTSEYNRKL---TEEEADKLEAFDFPALAVNLKIVKNA
OpaPAO3_W1Q987 -----------------------TTEKSVWNHPVLILNMFYISKV
KmaPAO_A0ABX6EWL ETVRNSDDLHHLLSTM--REKLPRSNDDSWKNPTYIVNLAKRTGI
LtePAO_C5DGS8 QRVREARDLSLLVEELDANTKYQFERGDSWNFPLLFVNMVKHTGI
KboPAO_XP_0647165. EIVRNSVDISALTNDLYNLDAQQEEN--CWKHPFYIINFQKVNGT
SlaPAO_From_genome ----------------------PEKPVRAFSQPVLFINMANVNGD
CjaPAO_A0A1E4SY1 QAIKTGSEI---------PDYPHSDKPKTWEHPVLFVNMATTQKI
406 410 412 414 416 418 420 422 424 426 428 430 432 434 436 438 440 442 444 446 450
FMS1_P50264 --ASFMMLMQAPLTNHIESIREDKERLF-SFFQPVLNKIMKCLDS
DhaPAO_A9E360 SKASLALLTQSPLTEYVES---HPEKAW-SYFKPMLSKLA-----
OpaPAO1_W1QA33 --PVLLALTSSPLTRYIES--HEIDDFW-PVFRPALANIANL---
OpaPAO2_W1QAC1 --PALMFLVPAPASHLLEE---NVEKYGPKLLEPIIAKLSGR---
OpaPAO3_W1Q987 --PSLVCFIQGRITEHLER---NPHLAW-EYFQPLLRKLG-----
KmaPAO_A0ABX6EWL --PTFVALTQQPLTEYLETLS--KEEVE-QFYRPVINKLLHSLDA
LtePAO_C5DGS8 --PSFVMLMANPLTKYIESMN-DKGKIF-EFFTPVLENLLKTLGC
KboPAO_XP_0647165. --PSLMLLTQEPVSKYIESLNGDYEKIF-QYFEPILNKVMSTFNS
SlaPAO_From_genome --ASLVAVTAGEMTLELES---HPEKAW-EVLKPAVETFCPGIG-
CjaPAO_A0A1E4SY1 --PSLVVLTQSPLTDYLES---NPQYAW-EYLRPMVLKLANA---
451 454 456 458 460 462 464 466 468 470 472 474 476 478 480 482 484 486 488 490 492 495
FMS1_P50264 EDVIDGM-RPIENIA---NANKP-VLRNIIVSNWTRDPYSRGAYS
DhaPAO_A9E360 ---ID-------------NKHVS-DPINTITSNWTNNPYIRGSYT
OpaPAO1_W1QA33 -----------------PDSCIP-RPTNVVTSNWSADPFARGSYL
OpaPAO2_W1QAC1 -----------------KQSELP-KPNLVFSTNWSSDPFSRGSVS
OpaPAO3_W1Q987 --------------------PLPLAPTNVIATSWTIDPFARGSYA
KmaPAO_A0ABX6EWL QDYI----CDLQDKIPDSDSEAP-VLKNIITTKWSSDPFALGAYS
LtePAO_C5DGS8 AEPIV---KDFENKLVKSNKKAP-ILKNIVTTNWTRDDYALGAYS
KboPAO_XP_0647165. GSVVNALNKDISEV-----GNHA-ILKNIICSNWSSDPFSRGSYT
SlaPAO_From_genome ------------------------DPVVVKASSWSVNPYFRGSYS
CjaPAO_A0A1E4SY1 -------------------EQVP-EPINVLVSPWTQDPYQRGSYT
496 500 502 504 506 508 510 512 514 516 518 520 522 524 526 528 530 532 534 536 540
FMS1_P50264 ACFPGDDPVDMVVAMSNGQD------SRIRFAGEHTIMDGAGCAY
DhaPAO_A9E360 AVHTGDDPLDLITQLSGEHGCGLSEKN-IRFAGEHTTIDGSGCVH
OpaPAO1_W1QA33 GCAVGDLIDEAIDAFTEAKD-VFDGKGRVRFVGEALIEEGNGCAH
OpaPAO2_W1QAC1 GNAVGD------VLVNDGLI---EGVNRLRFAGEAYCYEGHTGAH
OpaPAO3_W1Q987 ACRPGDDPTDVIINLERGFG------N-VRFAGEHTILDGAGAVH
KmaPAO_A0ABX6EWL ACKPGDDPMDLVIALSAGQG------N-LRFAGEHTIMDGAGCAY
LtePAO_C5DGS8 ACEPGDDPMDLVLALTENQT------SLVRFAGEHTIMDGAGCVY
KboPAO_XP_0647165. ACKPGDDGWDMVLAMNEGQD------SKIRFAGEHTVMDGAGCVY
SlaPAO_From_genome VSLVDDEPFMALEAMGKGVG------K-LRFAGEHTVWEGNGCVN
CjaPAO_A0A1E4SY1 ACHPGDDPLQSIIAFETGFG------N-VRFAGEHTILDGTGCVM

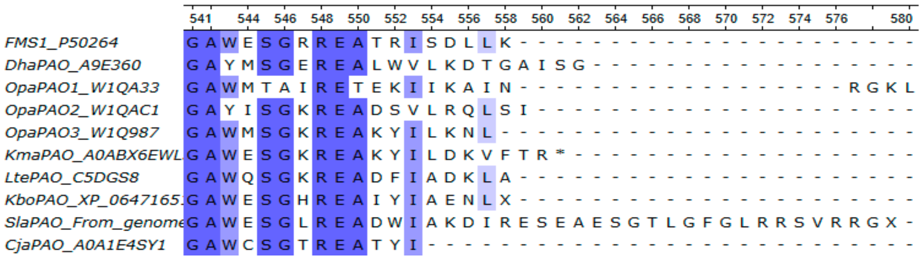

Figure S1. Multiple sequence alignment of known yeast polyamine oxidase and proteins, used in this study.

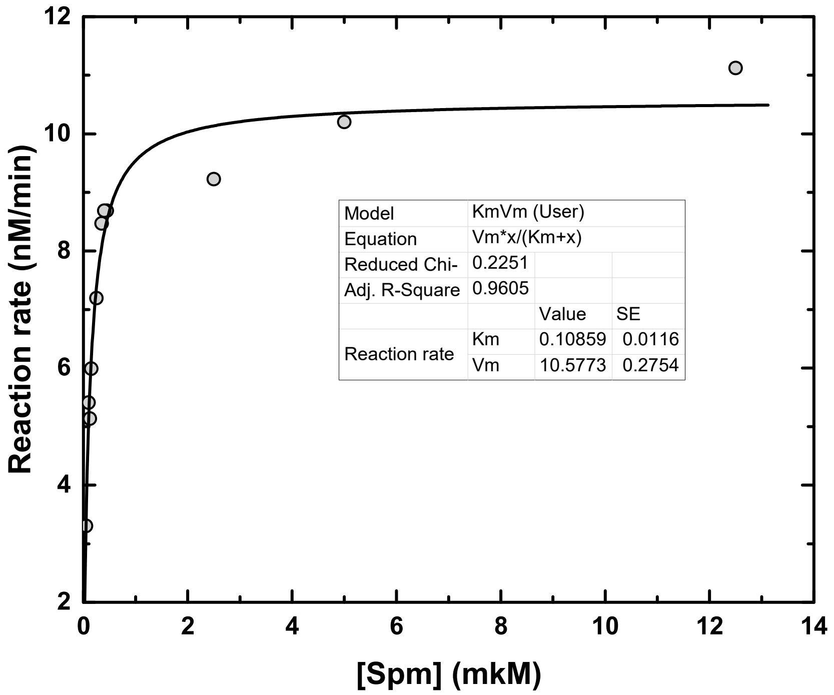

Figure S2. Michaelis–Menten kinetics of KmaPAO with spermine as the substrate. Initial reaction rates were measured at different spermine concentrations. The final KmaPAO concentration in the reaction mixture was 4.2 ng/mL. The experimental data were fitted to the Michaelis–Menten equation. Open circles represent the experimental values, and the solid line represents the nonlinear regression fit.

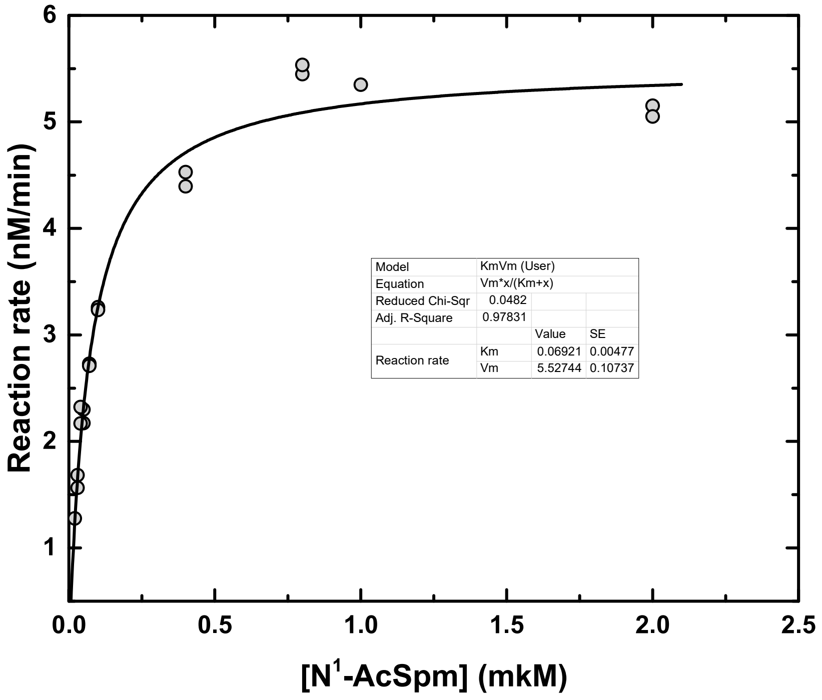

Figure S3. Michaelis–Menten kinetics of KmaPAO with N[1]-acetylspermine as the substrate. Initial reaction rates were measured at different N[1]-acetylspermine concentrations. The final KmaPAO concentration in the reaction mixture was 4.2 ng/mL. The experimental data were fitted to the Michaelis–Menten equation. Open circles represent the experimental values, and the solid line represents the nonlinear regression fit.

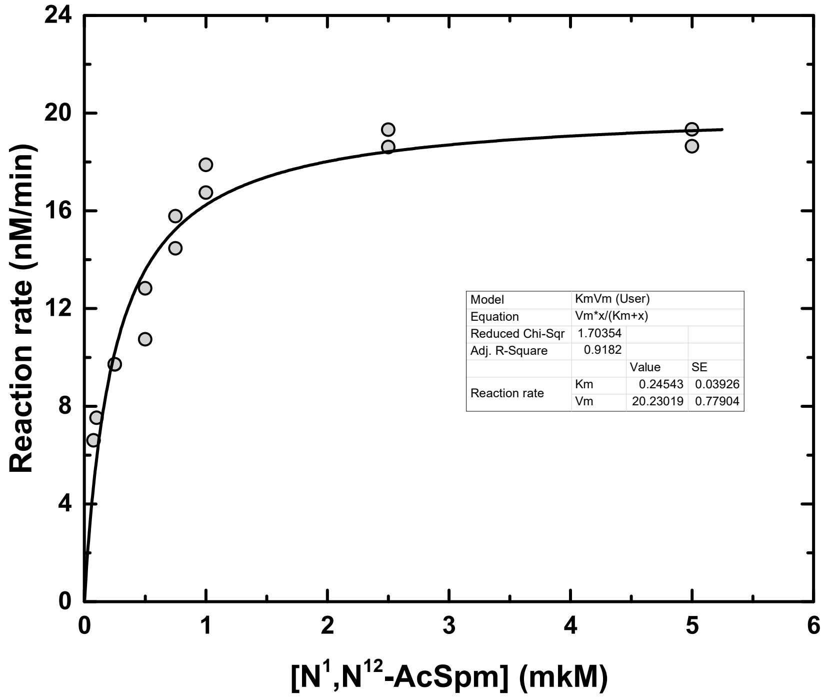

Figure S4. Michaelis–Menten kinetics of KmaPAO with N[1,12]-diacetylspermine as the substrate. Initial reaction rates were measured at different N[1,12]-diacetylspermine concentrations. The final KmaPAO concentration in the reaction mixture was 4.2 ng/mL. The experimental data were fitted to the Michaelis–Menten equation. Open circles represent the experimental values, and the solid line represents the nonlinear regression fit.

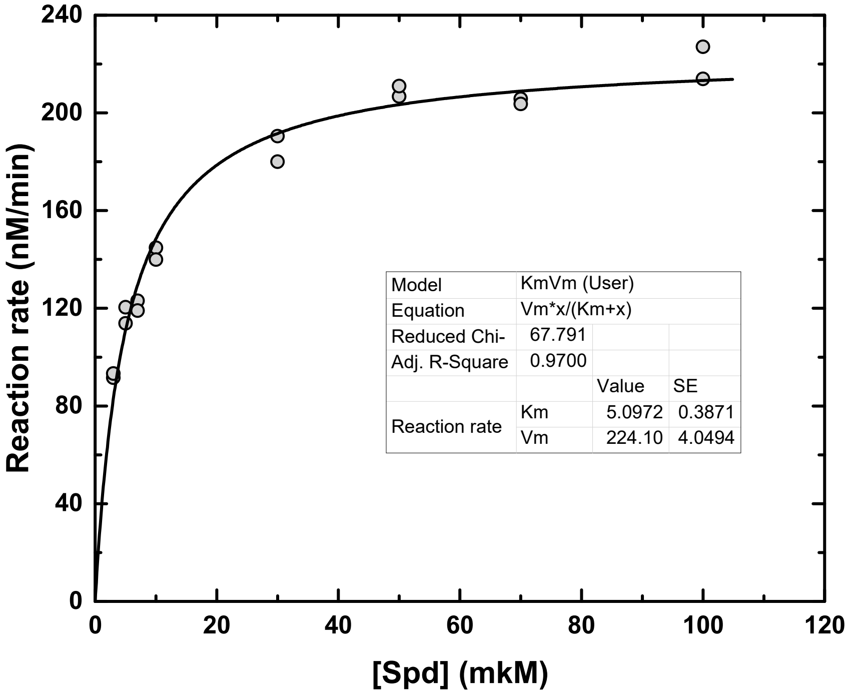

Figure S5. Michaelis–Menten kinetics of KmaPAO with spermidine as the substrate. Initial reaction rates were measured at different spermidine concentrations. The final KmaPAO concentration in the reaction mixture was 4.2 ng/mL. The experimental data were fitted to the Michaelis–Menten equation. Open circles represent the experimental values, and the solid line represents the nonlinear regression fit.

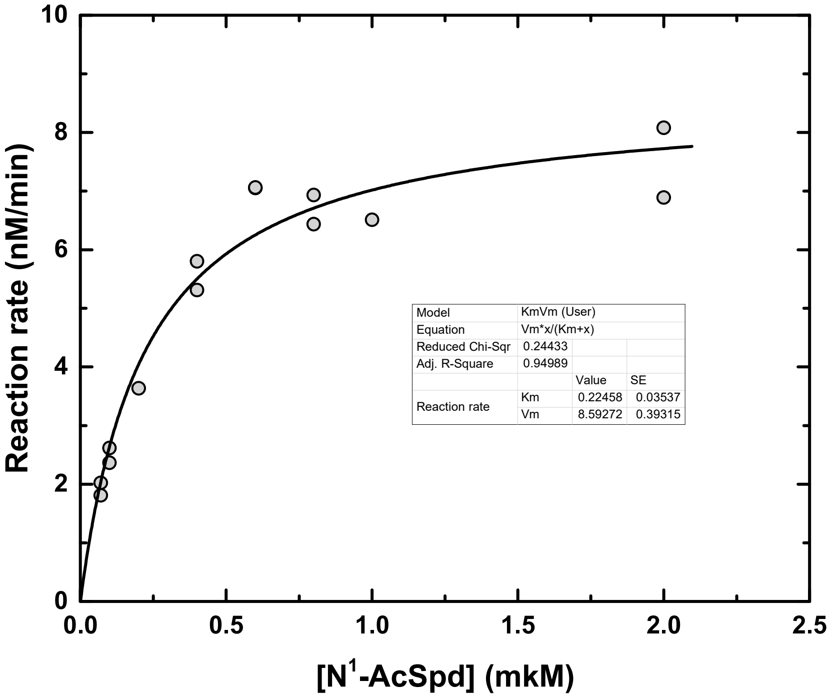

Figure S6. Michaelis–Menten kinetics of KmaPAO with N^1-acetylspermidine as the substrate. Initial reaction rates were measured at different N^1-acetylspermidine concentrations. The final KmaPAO concentration in the reaction mixture was 375 ng/mL. The experimental data were fitted to the Michaelis–Menten equation. Open circles represent the experimental values, and the solid line represents the nonlinear regression fit.

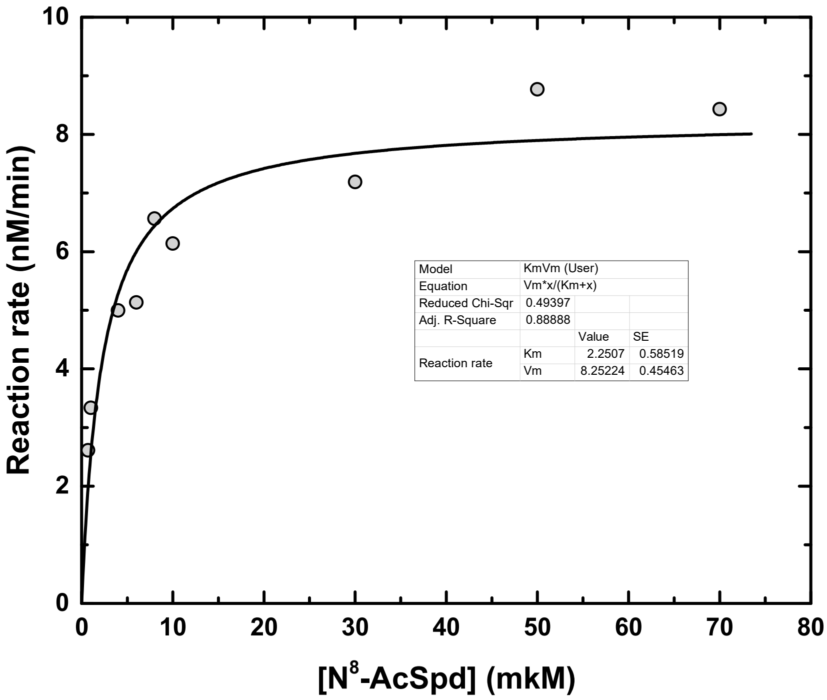

Figure S7. Michaelis–Menten kinetics of KmaPAO with N[8]-acetylspermidine as the substrate. Initial reaction rates were measured at different N[8]-acetylspermidine concentrations. The final KmaPAO concentration in the reaction mixture was 4.2 ng/mL. The experimental data were fitted to the Michaelis–Menten equation. Open circles represent the experimental values, and the solid line represents the nonlinear regression fit.
